# PTEN regulates colorectal epithelial apoptosis through Cdc42 signalling

**DOI:** 10.1038/bjc.2011.384

**Published:** 2011-09-27

**Authors:** R Deevi, A Fatehullah, I Jagan, M Nagaraju, V Bingham, F C Campbell

**Affiliations:** 1 Centre for Cancer Research and Cell Biology, Queen's University of Belfast, Lisburn Road, Belfast BT97BL, UK

**Keywords:** apoptosis, PTEN, Cdc42, PARP, GSK3*β*

## Abstract

**Background:**

Phosphatase and tensin homologue deleted on chromosome 10 (PTEN) regulation of the Rho-like GTPase Cdc42 has a central role in epithelial polarised growth, but effects of this molecular network on apoptosis remain unclear.

**Methods:**

To investigate the role of Cdc42 in PTEN-dependent cell death, we used flow cytometry, *in vitro* pull-down assays, poly(ADP ribose) polymerase (PARP) cleavage and other immunoblots in isogenic PTEN-expressing and -deficient colorectal cells (HCT116PTEN^+/+^, HCT116PTEN^−/−^, Caco2 and Caco2 ShPTEN cells) after transfection or treatment strategies.

**Results:**

The PTEN knockout or suppression by short hairpin RNA or small interfering RNA (siRNA) inhibited Cdc42 activity, PARP cleavage and/or apoptosis in flow cytometry assays. Transfection of cells with wild-type or constitutively active Cdc42 enhanced PARP cleavage, whereas siRNA silencing of Cdc42 inhibited PARP cleavage and/or apoptosis. Pharmacological upregulation of PTEN by sodium butyrate (NaBt) treatment enhanced Cdc42 activity, PARP cleavage and apoptosis, whereas Cdc42 siRNA suppressed NaBt-induced PARP cleavage. Cdc42-dependent signals can suppress glycogen synthase kinase-*β* (GSK3*β*) activity. Pharmacological inhibition of GSK3*β* by lithium chloride treatment mimicked effects of Cdc42 in promotion of PARP cleavage and/or apoptosis.

**Conclusion:**

Phosphatase and tensin homologue deleted on chromosome 10 may influence apoptosis in colorectal epithelium through Cdc42 signalling, thus providing a regulatory framework for both polarised growth and programmed cell death.

Phosphatase and tensin homologue deleted on chromosome 10 (PTEN) dephosphorylates intracellular phosphatidylinositol 3,4,5 trisphosphate (Maehama and Dixon, 1999) and thus antagonises the phosphatidylinositol 3-kinase/AKT pathway (Sun *et al*, 1999). The PTEN loss in cancer states invokes unopposed AKT activity and phosphorylation of various substrates that enhance cell survival, including pro-caspase 9 (Cardone *et al*, 1998), BAD (Datta *et al*, 1997) and the forkhead factor, FKHR (Biggs *et al*, 1999). Although therapeutic targeting of AKT could provide pro-apoptotic cancer therapy (Bailey *et al*, 2006; Chee *et al*, 2007; Yap *et al*, 2008), effects of small interfering RNA (siRNA) knockdown of AKT on apoptosis are inconsistent (Koseoglu *et al*, 2007) and results from recent phase I and II clinical trials of AKT inhibitor monotherapy have been disappointing (Argiris *et al*, 2006; Yap *et al*, 2008; Gills and Dennis, 2009). Biological consequences of PTEN loss and AKT activation do not overlap completely (Lee *et al*, 2010), and deeper understanding of PTEN-regulated cell death may help delineate novel therapeutic strategies.

In physiological conditions, PTEN maintains tissue homeostasis by coordinated regulation of polarised cell growth (Kolsch *et al*, 2008) and morphogenesis (Martin-Belmonte *et al*, 2007), as well as cell death (Weng *et al*, 2001). The Rho-like GTP-binding protein Cdc42 (Cell division cycle 42) is a key mediator of PTEN-dependent cell polarisation (Higuchi *et al*, 2001) and morphogenesis (Martin-Belmonte *et al*, 2007). Cdc42 cycles between active GTP-bound and inactive GDP-bound states and interacts with highly conserved polarity proteins partitioning defective homologue 6 (Par6) and atypical protein kinase C (aPKC). The resulting Cdc42/Par6/aPKC complex deactivates glycogen synthase kinase-3*β* (GSK3*β*) by promotion of serine 9 (Ser9) phosphorylation (Etienne-Manneville and Hall, 2003). The GSK3*β* modulates microtubule dynamics implicated in cell polarisation processes (Zhou and Snider, 2005).

In addition to its effects on cell polarisation (Johnson, 1999; Etienne-Manneville, 2004), Cdc42-dependent signalling also influences growth (Cerione, 2004) and apoptosis (Chuang *et al*, 1997; Na *et al*, 1999; Melendez *et al*, 2011). As a target of the Cdc42/Par6/aPKC complex (Etienne-Manneville and Hall, 2003), GSK3*β* integrates multiple signalling cascades (Doble and Woodgett, 2003) and has context-specific effects on apoptosis (Pap and Cooper, 1998; Hoeflich *et al*, 2000; Beurel and Jope, 2006). Glycogen synthase kinase-3*β* enhances survival of gastrointestinal cancer cells (Mai *et al*, 2009), and GSK3*β* knockout mice succumb to massive hepatic apoptosis (Hoeflich *et al*, 2000), indicating an important anti-apoptotic role.

Although PTEN influences tissue homeostasis through Cdc42 signalling (Langlois *et al*, 2010), the role of Cdc42 activity in PTEN-dependent cell death remains unclear. To investigate the role of PTEN in Cdc42-mediated apoptosis, we used paired isogenic human colorectal cancer (CRC) cell lines differing in PTEN status. We used parental HCT116PTEN^+/+^ cells and a knockout clone generated by a high-efficiency promoterless PTEN targeting vector (Lee *et al*, 2004). To address the phenotypic and genotypic heterogeneity of human CRC, we generated a second unrelated isogenic human colorectal model with genotype and phenotype differences from the HCT116 system. We used a Caco-2 parental cell line that expresses PTEN (Wang *et al,* 2007). Unlike HCT116 cells, Caco-2 cells are K-Ras wild type (wt), but APC mutant (Ilyas *et al,* 1997; Lawson *et al*, 2000; Hao *et al*, 2007; Database, 2011), and undergo apico-basolateral polarisation (Gilbert *et al*, 1991).

Here, we show that PTEN knockout in HCT116 colorectal cells or suppression by stable short hairpin RNA (shRNA) transfection in Caco2 colorectal cells inhibits Cdc42 activity, poly(ADP ribose) polymerase (PARP) cleavage and/or apoptosis in flow cytometry assays of propidium iodide-labelled cells (PI-labelled apoptosis). Sodium butyrate (NaBt) is a short-chain fatty acid that upregulates PTEN expression (Wang *et al*, 2001; Bai *et al*, 2010). In this study, NaBt treatment enhanced PTEN expression, Cdc42 activity and apoptosis, whereas siRNA knockdown of Cdc42 impeded NaBt-induced cell death. Lithium chloride (LiCl) treatment suppresses GSK3*β* (De Sarno *et al*, 2002). In the present study, LiCl treatment mimicked effects of Cdc42 in promotion of GSK3*β* Ser9 phosphorylation, PARP cleavage and apoptosis. Taken together, these data implicate Cdc42 signalling in PTEN regulation of apoptosis, in genotypically and phenotypically distinct colorectal epithelial lines.

## Materials And Methods

### Reagents and antibodies

Laboratory chemicals were purchased from Sigma-Aldrich (Poole, UK), unless otherwise stated. GeneJuice transfection reagent was purchased from Novagen (Gibbstown, NJ, USA). Rabbit monoclonal anti-PTEN, anti-GSK3*β* and -p-GSK3*β* (Ser9) primary antibodies were purchased from Cell Signaling Technology (New England Biolabs Ltd, Hitchin, Herts, UK). Mouse monoclonal (MMC) anti-Cdc42 was obtained from BD Transduction Laboratories (San Diego, CA, USA), and MMC anti-GAPDH was obtained from Abcam (Cambridge, MA, USA). Depending on protein expression levels, LICOR (Infra-Red imaging system; LICOR Biosciences, Cambridge, UK) or enhanced chemiluminescence (Amersham, GE Healthcare, Buckinghamshire, UK) detection systems were used. Primary antibodies were therefore used with anti-rabbit LICOR IRDye 680, anti-mouse LICOR IRDye800 or horseradish peroxidase-conjugated anti-rabbit or anti-mouse secondary antibodies for western blotting. pcDNA3-Cdc42-wt (no. 12599), Cdc42 Q61L (constitutively active (CA); no. 12600), Cdc42 T17N (dominant negative (DN); no.12601), pcDNA-EGFP empty vector (EV), pMKO.1 puro PTEN shRNA (no. 10669) and pMKO.1 puro EV (no. 8452) were purchased from Addgene Inc., Cambridge, MA, USA. Lithium chloride and NaBt were dissolved in distilled water immediately before use.

### Cell culture and treatments

HCT116PTEN^−/−^ and HCT116PTEN^+/+^ cells were cultured in McCoy's 5A media (Sigma-Aldrich, Dorset, UK) supplemented with 10% fetal calf serum (FCS), 1 mM L-glutamine, 1 mM sodium pyruvate and supplemented with antibiotics. Caco2 ShPTEN and Caco2 cells were cultured in Eagle's minimal essential medium supplemented with 10% FCS, 1 mM non-essential amino acids, 1 mM L-glutamine and antibiotics at 37° C in 5% CO_2_. Parental cells and clones have been passaged no more than 20 times, in accord with UKCCCR Guidelines (UKCCCR, 2000).

Cells were grown for 2–3 days until 60–70% confluence and then treated with the GSK3*β* inhibitor LiCl 1 mM (De Sarno *et al*, 2002) or NaBt 1 mM (Wang *et al*, 2001) for 24 h. Drug-treated or -untreated cells were then lysed with ice-cold lysis buffer for 15 min. Cell lysate was collected, vortexed for 1 min and then centrifuged at 12 400 g for 10 min at 4 °C. Protein concentration was measured by the bicinchoninic acid kit using bovine serum albumin standards (Thermo Scientific, Barrington, IL, USA).

### Cell transfection

HCT116PTEN^−/−^, HCT116PTEN^+/+^, Caco2 ShPTEN and Caco2 cells were plated at 1.5. × 10^5^ cells per well in six-well plates and incubated for 24 h at 37° C, and then transfected with 600 ng DNA per well for all constructs. We used GeneJuice transfection reagent/serum-free medium/DNA mix, according to manufacturer's instructions. Cells were incubated with DNA-GeneJuice complexes for 24 h. Transfection medium was then replaced with normal culture medium. Cells were collected 48 h after transfection for PI staining and flow cytometry or lysed and probed as described in *Protein extraction and western blotting.*

### Stable retroviral transfections

Caco2 cells were transfected using replication-defective retroviral vectors encoding PTEN shRNA or EV only in a puromycin selection cassette, as previously described (Boehm *et al*, 2005). Retroviral vectors were generated using the Phoenix retroviral expression system (Orbigen, San Diego, CA, USA; Geng *et al*, 2010). Briefly, Phoenix Eco retroviral packaging cells (Orbigen), purchased within the last 2 years, were transfected with pMKO.1 puro PTEN shRNA or pMKO.1 puro EV retroviral expression vectors (Addgene Inc.), and supernatant containing recombinant retroviral vectors encoding PTEN shRNA or EV only was collected after 48 h. Viral supernatant was centrifuged at 2000 g at room temperature for 15 min to remove cell debris and then used immediately. Viral supernatant was added to Caco2 cultures for 48 h at 37° C in 5% CO_2_. Caco2 transfectants were then incubated in 1 *μ*g ml^−1^ puromycin for 7 days for selection of ShPTEN- or EV-only-positive subclones. HCT116PTEN^−/−^ cells and HCT116PTEN^+/+^ cells were a gift from Dr Tod Waldman, Georgetown University, USA. The HCT116PTEN^−/−^ knockout clone was generated from parental cells using a high-efficiency promoterless PTEN targeting vector, as previously described (Lee *et al*, 2004).

### Small interfering RNA-mediated knockdown of PTEN

RNA interference-mediated knockdown of PTEN was performed by transfection of synthetic duplex RNA oligonucleotides, using Oligofectamine (Invitrogen, Carlsbad, CA, USA) according to manufacturers’ instructions. On-Target plus SMART pool PTEN (catalog no. L-003023-00, Dharmacon, Fisher Scientific, Dublin, UK) and Cdc42 (catalog no. L-005057 from Dharmacon) siRNA oligonucleotides were used. On-Target plus non-targeting siRNAs were used as controls (catalog no. D-001810-01-05, Dharmacon). Briefly, Oligofectamine and Opti-Mem serum-free medium (Invitrogen) were mixed in 1 : 3 ratio, incubated at room temperature for 5 min and then added to siRNA, which was prediluted in Opti-Mem to final concentration of 50 nM and then added to cultures. Cells were grown in 90 mm dishes and were transfected at 50–60% confluence by incubation in the above mixture. The medium was changed after 6 h and cells were harvested after 48 h. The PTEN or Cdc42 expression was assayed by western blot as outlined in *Protein extraction and western blotting.*

### GST–PAK pull-down assay for Cdc42 activity

Cells were lysed in buffer comprising 50 mM Tris-HCl, pH 7.5, 1% Triton X-100, 100 mM NaCl, 10 mM MgCl_2_, 5% glycerol, 1 mM Na_3_VO_4_ and protease inhibitor cocktail (Roche, West Sussex, UK). Lysate was centrifuged at 12 500 g for 10 min. p21-activated kinase (PAK) binds specifically to active Cdc42 and Rac (Chuang *et al*, 1997). The GTP-bound form of endogenous or GFP-tagged Cdc42 was assayed by adding GST–PAK fusion protein coupled with glutathione sepharose 4B beads (GE Healthcare, Svensk, Sweden) to 1 mg of cell lysate and then incubated at 4° C on a rotating wheel for 1 h. Beads were centrifuged for 1 min, washed three times and then resuspended in 40 *μ*l of 2 × Laemmli buffer containing 3 *μ*l of 1 M DTT. Cdc42 activity was then assayed by western blotting, as outlined below. Experiments were repeated in triplicate.

### Protein extraction and western blotting

Protocols were the same as we have previously described for other proteins (El-Tanani *et al*, 2004). Briefly, proteins were resolved using gel electrophoresis, followed by blotting onto nitrocellulose membranes. Membranes were probed using antibodies as indicated in the text. Experiments were repeated in triplicate.

### Apoptosis assays

Cell death was assessed by PARP cleavage assay, as we have previously described (Collett and Campbell, 2006), and by flow cytometry, using PI for nuclear staining, to enable assay of the hypodiploid sub-G0/G1 peak (PI-labelled apoptosis; Riccardi and Nicoletti, 2006).

### Densitometry and statistical analysis

Densitometric analysis of western blots was carried out using ImageJ (Image processing and analysis in JAVA; NIH public domain software). Data analysis was carried out by ANOVA using SPSS (v16) for Windows (IBM Corp., New York, NY, USA). Descriptive statistics were expressed as the mean±s.e.m. Graphs were drawn using GraphPad Prism 5 (La Jolla, CA, USA).

## Results

### PTEN expression associates with enhanced Cdc42 activation, increased PARP cleavage and apoptosis

**Figure 1:**
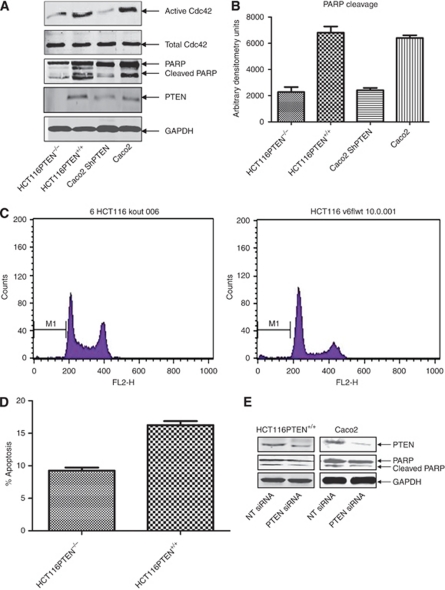
Phosphatase and tensin homologue deleted on chromosome 10 regulates apoptosis through activation of Cdc42 in colorectal epithelial cells. (**A**) Cdc42 activity and PARP cleavage in PTEN-deficient or -expressing colorectal cells. HCT116PTEN^−/−^, HCT116PTEN^+/+^, Caco2 ShPTEN and Caco2 colorectal epithelial cells were lysed, immunoblotted with anti-PTEN, anti-PARP, anti-Cdc42 and anti-GAPDH. Active Cdc42 was assessed by GST–PAK pull-down assay (*n*=3). (**B**) Summary effects of PTEN expression on PARP cleavage. Densitometry assays showed lower PARP cleavage in PTEN-deficient HCT116PTEN^−/−^ and Caco2 ShPTEN cells than in parental HCT116PTEN^+/+^ or Caco2 cells, respectively (*P*<0.001; ANOVA; *n*=3). (**C**) Effects of PTEN expression on PI-labelled apoptosis in HCT116 clones. DNA histogram of the sub-G0/G1 fraction of PI-stained cells (region M1) by flow cytometry after elimination of residual debris. By this method, apoptosis was quantified as 9.35±0.96% *vs* 16.75±1.5% in HCT116PTEN^−/−^ *vs* HCT116PTEN^+/+^ cells (*P*<0.001; ANOVA). (**D**) Summary effects of PTEN expression on PI-labelled apoptosis in HCT116 clones. Propidium iodide-labelled apoptosis (% Apoptosis) was lower in HCT116PTEN^−/−^ than in HCT116PTEN^+/+^ cells (*n*=3; *P*<0.001; ANOVA). (**E**) Effects of PTEN siRNA knockdown on PARP cleavage. Small interfering RNA knockdown of PTEN in HCT116PTEN^+/+^ or Caco2 cells suppresses PARP cleavage.

To investigate PTEN regulation of Cdc42 and apoptosis, we used a PTEN knockout isogenic subclone of HCT116 cells together with parental cells (HCT116PTEN^−/−^ and HCT116PTEN^+/+^ cells, respectively). We also raised an unrelated PTEN-deficient subclone of Caco2 cells by PTEN shRNA stable transfection (Caco2 ShPTEN cells). Cdc42 activation was assessed by GST–PAK pull-down assay, whereas cell death was assayed by PARP cleavage immunoblots, as well as PI labelling and flow cytometry. The PTEN knockout or suppression was associated with lower Cdc42 activation and PARP cleavage in HCT116PTEN^−/−^ and Caco2 ShPTEN cells than in parental HCT116PTEN^+/+^ and Caco2 cells, respectively (Figures 1a and b). Flow cytometry assays were conducted in HCT116 clones only. Analysis of PI-stained cells after gating of debris or necrotic cells showed apoptosis in 9.25±0.98% *vs* 16.75±1.5% in HCT116PTEN^−/−^ *vs* HCT116PTEN^+/+^ cells (Figures 1c and d). Transfection of HCT116PTEN^+/+^ or Caco2 cells with PTEN siRNA suppressed PARP cleavage (Figure 1e).

### Cdc42 is implicated in PTEN-dependent cell death

**Figure 2:**
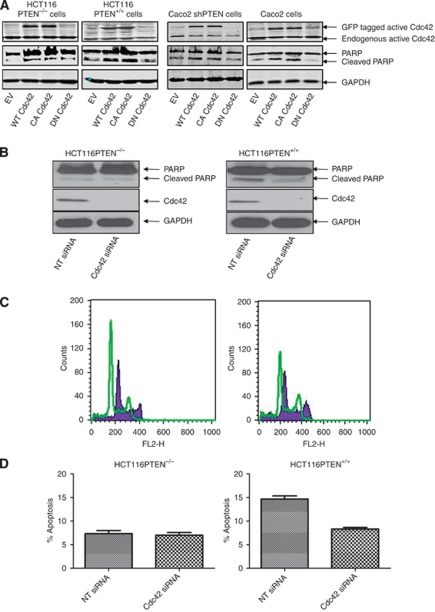
Cdc42 mediates PTEN-dependent apoptosis in epithelial cells. (**A**) Effects of Cdc42 transfection on PARP cleavage. HCT116PTEN^−/−^, HCT116PTEN^+/+^, Caco2 ShPTEN and Caco2 cells were transfected with GFP-tagged EV-only, wt, CA and DN Cdc42 in expression vectors. The GFP-tagged transfected and endogenous Cdc42 activity and PARP cleavage are shown. The GFP-tagged Cdc42 activity and PARP cleavage are enhanced by wt and CA Cdc42 transfections. (**B**) Effects of Cdc42 siRNA knockdown on PARP cleavage. Transfection of HCT116PTEN^−/−^ and HCT116PTEN^+/+^ cells with scrambled non-targeting (NT) siRNA or Cdc42-targeting siRNA (Cdc42 siRNA). Cdc42 siRNA suppressed PARP cleavage in HCT116PTEN^+/+^ cells. (**C**) Effects of Cdc42 siRNA knockdown on PI-labelled apoptosis. DNA histograms of the sub-G0/G1 fraction of PI-stained HCT116PTEN^−/−^cells (left panel) and HCT116PTEN^+/+^ cells (right panel) after transfection with NT siRNA (solid histogram) of Cdc42 siRNA (overlay). Transfection of Cdc42 siRNA depresses the sub-G0/G1 apoptotic peak in HCT116PTEN^+/+^ cells, in flow cytometry assays. (**D**) Summary effects of Cdc42 siRNA knockdown on PI-labelled apoptosis. Transfection with Cdc42-targeting siRNA (Cdc42 siRNA) inhibited PI-labelled apoptosis in HCT116PTEN^+/+^ cells (*n*=3; *P*<0.001; ANOVA).

To investigate the role of Cdc42 in PTEN-dependent cell death, we transfected cells with wt, CA or DN Cdc42 constructs. Transfection of wt and CA Cdc42 increased the levels of active Cdc42 and PARP cleavage in all cell types (Figure 2a). Although effects of DN Cdc42 appeared inconsistent (Figure 2a), silencing of Cdc42 by siRNA transfection inhibited PARP cleavage (Figure 2b) and PI-labelled apoptosis in HCT116PTEN^+/+^ cells (Figures 2c and d). Taken together, these data implicate Cdc42 in PTEN-dependent cell death in colorectal epithelium.

### NaBt treatment induces PTEN-dependent Cdc42 activation, PARP cleavage and PI-labelled apoptosis

**Figure 3:**
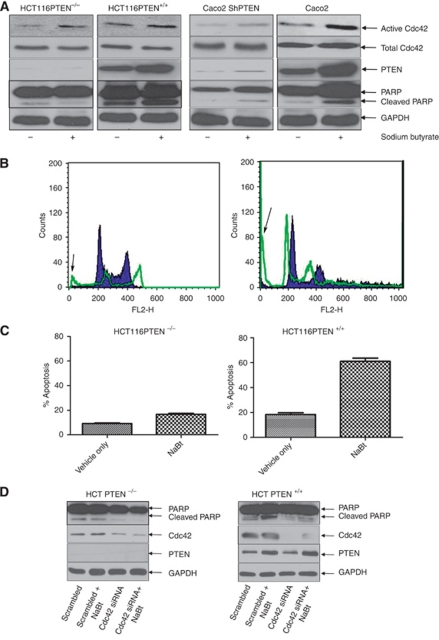
Effects of NaBt treatment. (**A**) Effects of NaBt treatment on PTEN, Cdc42 and PARP cleavage. HCT116PTEN^−/−^, HCT116PTEN^+/+^, Caco2 ShPTEN and Caco2 cells were treated by medium only or NaBt, then lysed and assayed for PTEN expression, Cdc42 activity and PARP cleavage. Sodium butyrate treatment upregulated PTEN, enhanced Cdc42 activity and promoted PARP cleavage in HCT116PTEN^+/+^ and Caco2 cells. Sodium butyrate treatment effects on Cdc42 activity and PARP cleavage were blunted by PTEN knockout or deficiency in HCT116PTEN^−/−^ and Caco2 ShPTEN cells, respectively. (**B**) Effects of NaBt treatment on PI-labelled apoptosis. DNA histograms of the sub-G0/G1 fraction of PI-labelled apoptotic cell death in HCT116PTEN^−/−^ cells (left panel) or HCT116PTEN^+/+^ cells (right panel) after treatment by vehicle only (solid histogram) or NaBt (overlay). Arrows denote sub-G0/G1 apoptosis peaks induced by NaBt treatment in HCT116PTEN^−/−^ and HCT116PTEN^+/+^ cells in flow cytometry assays. (**C**) Summary effects of NaBt treatment on PI-labelled apoptosis. Sodium butyrate treatment enhanced PI-labelled apoptosis in HCT116PTEN^+/+^ cells (*P*<0.001; ANOVA) but was less effective in HCT116PTEN^−/−^ cells. (**D**) Effects of Cdc42 SiRNA knockdown on NaBt-induced PARP cleavage. HCT116PTEN^−/−^ and HCT116PTEN^+/+^ cells were transfected with NT or Cdc42 siRNA and treated with vehicle only or NaBt. Cdc42, PARP and GAPDH were detected by probing with corresponding anti-primary antibodies, followed by appropriate horseradish peroxidase-conjugated secondary antibodies.

Sodium butyrate is a short-chain fatty acid that upregulates PTEN (Wang *et al*, 2007) and induces apoptosis in colorectal epithelium (Hague and Paraskeva, 1995). The NaBt treatment enhanced PTEN expression, Cdc42 activation and PARP cleavage in HCT116PTEN^+/+^ and Caco2 cells and significantly enhanced PI-labelled apoptosis in HCT116PTEN^+/+^ cells. Conversely, PTEN knockout or deficiency attenuated NaBt treatment effects on Cdc42 activity, PARP cleavage and/or PI-labelled apoptosis (Figures 3a–c). Independent and interactive effects of PTEN expression and NaBt treatment on PI-labelled apoptosis were significant (*P*<0.001; two-way ANOVA). To investigate the role of Cdc42 in NaBt-induced cell PARP cleavage, we conducted Cdc42 siRNA knockdown experiments. Cdc42 siRNA inhibited Cdc42 expression and PARP cleavage in vehicle-only- and NaBt-treated HCT116PTEN^−/−^ and HCT116PTEN^+/+^ cells (Figure 3d).

### Pharmacological suppression of GSK3*β* signalling

**Figure 4:**
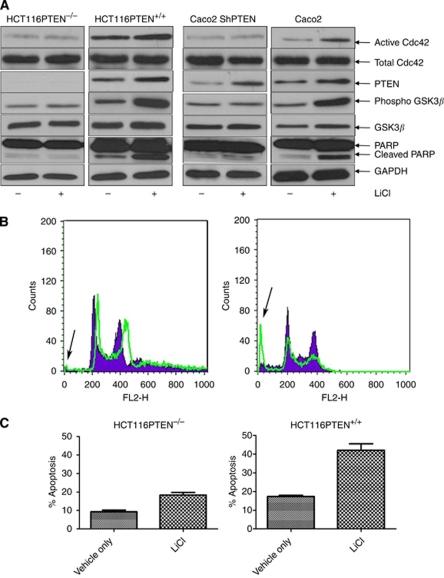
Effects of pharmacological suppression of GSK3*β*. (**A**) Effects of LiCl treatment on PARP cleavage. HCT116PTEN^−/−^, HCT116PTEN^+/+^, Caco2 ShPTEN and Caco2 cells were treated with vehicle only (−) or LiCl (1 mM; +). The LiCl treatment enhanced GSK3*β* Ser9 phosphorylation and PARP cleavage, to a greater extent in HCT116PTEN^+/+^ and Caco2 cells than in HCT116PTEN^−/−^ or Caco2 ShPTEN cells. The LiCl treatment also enhanced PTEN expression and Cdc42 activation in HCT116PTEN^+/+^ and Caco2 cells. (**B**) Effects of LiCl treatment on PI-labelled apoptosis. DNA histograms of the sub-G0/G1 fraction of PI-labelled apoptotic cell death in HCT116PTEN^−/−^ cells (left panel) and HCT116PTEN^+/+^ cells (right panel) after treatment by vehicle only (solid histogram) or LiCl (overlay). Arrows denote sub-G0/G1 apoptosis peaks induced by LiCl in HCT116PTEN^−/−^ and HCT116PTEN^+/+^ cells, in flow cytometry assays. (**C**) Summary effects of LiCl treatment on PI-labelled apoptosis in HCT116PTEN^−/−^ or HCT116PTEN^+/+^ cells. Interactive effects of LiCl treatment and PTEN status on PI-labelled apoptosis (% Apoptosis) were significant (*P*<0.001; *n*=3).

As Cdc42-dependent signals may suppress GSK3*β* (Etienne-Manneville and Hall, 2003), a context-specific regulator of apoptosis (Beurel and Jope, 2006), we used pharmacological inhibitor studies to explore the role of GSK3*β* in apoptosis of PTEN-expressing or -deficient colorectal cells. Treatment of cells with the GSK3*β* inhibitor, LiCl, promoted GSK3*β* Ser9 phosphorylation and enhanced PARP cleavage. These effects were reduced by PTEN knockout or deficiency in HCT116PTEN^−/−^ or Caco2 ShPTEN cells, respectively (Figure 4a). Similarly in flow cytometry assays, LiCl enhanced PI-labelled apoptosis in HCT116PTEN^+/+^ cells but effects were diminished by PTEN knockout in HCT116PTEN^−/−^ cells (Figures 4b and c). Lithium chloride treatment also enhanced PTEN expression and Cdc42 activity in PTEN-expressing cell lines (Figure 4a). Interactive effects of LiCl treatment and PTEN status on PI-labelled apoptosis were significant (*P*<0.001; two-way ANOVA), which may suggest involvement of GSK3*β* in PTEN/Cdc42-dependent cell death.

## Discussion

Phosphatase and tensin homologue deleted on chromosome 10 expression in murine fibroblasts can be associated with suppression of GTP-bound Cdc42 (Liliental *et al*, 2000). Conversely, PTEN promotes membrane recruitment and activation of Cdc42, whereas siRNA PTEN knockdown suppresses Cdc42 activity in MDCK epithelial cultures (Martin-Belmonte *et al*, 2007). Hence, PTEN may coordinate Cdc42 signalling through cell- or context-specific mechanisms. In the present study, we found higher Cdc42 activity in PTEN-expressing colorectal epithelial cells than in PTEN-null or -deficient clones. Furthermore, siRNA PTEN knockdown in parental cells inhibited Cdc42 activation. In accord with its pro-apoptotic role (Lu *et al*, 1999; Weng *et al*, 2001), we found that PTEN deletion or suppression in HCT116PTEN^−/−^ or Caco2 ShPTEN cells or siRNA knockdown of PTEN in HCT116PTEN^+/+^ and Caco2 cells inhibited PARP cleavage. Furthermore, flow cytometry analysis of the sub-G0/G1 fraction of cells, after PI staining and appropriate gating of debris and/or necrotic cells showed, reduced apoptosis in HCT116PTEN^−/−^ *vs* HCT116PTEN^+/+^ cells.

We conducted transfection studies to investigate the role of Cdc42 signalling in PTEN-mediated apoptosis. Transfection of cells with wt or CA Cdc42 enhanced Cdc42 activity and PARP cleavage, but effects of DN Cdc42 appeared less consistent. As DN Cdc42 mutants may have non-target effects by sequestering GEFs for different Rho-family GTPases (Feig, 1999; Wang *et al*, 2003), we also silenced Cdc42 by siRNA knockdown. This step suppressed PARP cleavage and PI-labelled apoptosis in HCT116PTEN^+/+^ cells. In addition to siRNA methods, we also used pharmacological strategies. Sodium butyrate enhances PTEN-mediated apoptosis in colorectal epithelium (Wang *et al*, 2007). In the present study, we found that NaBt treatment substantively enhanced PTEN expression, Cdc42 activation, PARP cleavage and or PI-labelled apoptosis in HCT116PTEN^+/+^ and/or Caco2 cells. Conversely, NaBt effects on PARP cleavage and PI-labelled apoptosis were attenuated by PTEN deletion or deficiency, in HCT116PTEN^−/−^ or Caco2 ShPTEN cells respectively. Furthermore, siRNA knockdown of Cdc42 suppressed NaBt induced PARP cleavage. Although NaBt may have off-target effects involving histone deacetylase inhibition (Walker *et al*, 2001) or vitamin D receptor activation (Gaschott and Stein, 2003), these pathways are not known to have a key role in Cdc42 activation. Taken together, these data implicate both PTEN and Cdc42 in NaBt-induced apoptotic cell death.

Cdc42-regulated signal transduction activates polarity proteins Par6 and aPKC (Cau and Hall, 2005; Schlessinger *et al*, 2007), which may regulate cell death through GSK3*β* (Kim *et al*, 2007). Although it is CA in resting cells (Frame and Cohen, 2001; Jope and Johnson, 2004), GSK3*β* may be inactivated by phosphorylation at Ser9 by AKT kinase activity (Cross *et al*, 1995) or by the Cdc42/Par6/aPKC polarisation complex (Etienne-Manneville and Hall, 2003). However, functional redundancy of AKT suppression of GSK3*β* has been shown in HCT116 cells by knockout studies (Ericson *et al*, 2010). Caco2 cells retain strong Cdc42 activity (Koch *et al*, 2009) that could influence their cell-specific GSK3*β* phosphorylation status (Etienne-Manneville and Hall, 2003). In the present study, we found that PTEN-expressing HCT116PTEN^+/+^ and Caco2 cells had low-level AKT activity (data not shown) but higher Cdc42 activity and GSK3*β* Ser9 phosphorylation *vs* HCT116PTEN^−/−^ and Caco2 ShPTEN cells.

As GSK3*β* has context-specific effects on apoptosis (Beurel and Jope, 2006), we investigated its role in PTEN-dependent cell death in HCT116 or Caco2 clones by LiCl pharmacological inhibition (De Sarno *et al*, 2002). Although LiCl may have off-targets effects, its predominant known function in colorectal epithelium is a Wnt mimetic and suppressor of GSK3*β* (Dihlmann *et al*, 2003). In the present study, LiCl treatment enhanced GSK3*β* Ser9 phosphorylation, increased PARP cleavage and PI-labelled apoptosis, thus indicating an anti-apoptotic role for GSK3*β* in the colorectal epithelial model systems. Furthermore, effects of LiCl on GSK3*β* Ser9 phosphorylation, PARP cleavage and apoptosis were attenuated by PTEN knockout or suppression in HCT116PTEN^−/−^ and Caco2 ShPTEN cells, indicating synergy between PTEN expression and LiCl treatment. We also found that LiCl treatment enhanced PTEN expression and Cdc42 activity in HCT116PTEN^+/+^ and Caco2 cells. These findings accord with previous studies that have shown that GSK3*β* destabilises PTEN by promotion of PTEN phosphorylation at Thr 366 (Al-Khouri *et al*, 2005) and that PTEN expression may be enhanced by GSK3*β* inhibitor treatment (Maccario *et al*, 2007). Phosphatase and tensin homologue deleted on chromosome 10 expression and LiCl treatment may thus share a common pathway of effect involving Cdc42 activation and GSK3*β* Ser9 phosphorylation in promotion of PARP cleavage and apoptosis.

Previous studies have shown regulatory coupling of cytoskeleton integrity and cell death by PTEN- (Vitolo *et al*, 2009; Tang *et al*, 2011) and Cdc42-dependent polarisation signalling (Warner *et al*, 2010). These processes may be implicated in eradication of cells that detach from supporting matrix or tissues (Cheng *et al*, 2004; Vitolo *et al*, 2009; Liu *et al*, 2011). In the present study, we show that PTEN-mediated PARP cleavage and apoptosis involves Cdc42 activity and may involve GSK3*β* Ser9 phosphorylation. Loss of these activities during cancer development may be implicated in unrestrained growth and formation of viable colonies from detached, depolarised neoplastic cells during tumour progression.
